# When Virtual Assistants Meet Teledermatology: Validation of a Virtual Assistant to Improve the Quality of Life of Psoriatic Patients

**DOI:** 10.3390/ijerph192114527

**Published:** 2022-11-05

**Authors:** Surya Roca, Manuel Almenara, Yolanda Gilaberte, Tamara Gracia-Cazaña, Ana M. Morales Callaghan, Daniel Murciano, José García, Álvaro Alesanco

**Affiliations:** 1 Aragón Institute of Engineering Research (I3A), University of Zaragoza, 50018 Zaragoza, Spain; 2 Department of Dermatology, Miguel Servet University Hospital, IIS Aragon, 50009 Zaragoza, Spain

**Keywords:** chat-based interaction, chronic patient support, eHeath, health information technology, mHealth, monitoring virtual assistant, psoriasis, quality of life, remote consultation, teledermatology

## Abstract

Teledermatology has given dermatologists a tool to track patients’ responses to therapy using images. Virtual assistants, the programs that interact with users through text or voice messages, could be used in teledermatology to enhance the interaction of the tool with the patients and healthcare professionals and the overall impact of the medication and quality of life of patients. As such, this work aimed to investigate the effectiveness of using a virtual assistant for teledermatology and its impact on the quality of life. We conducted surveys with the participants and measured the usability of the system with the System Usability Scale (SUS). A total of 34 participants (30 patients diagnosed with moderate-severe psoriasis and 4 healthcare professionals) were included in the study. The measurement of the improvement of quality of life was done by analyzing Psoriasis Quality of Life (PSOLIFE) and Dermatology Life Quality Index (DLQI) questionnaires. The results showed that, on average, the quality of life improved (from 63.8 to 64.8 for PSOLIFE (with a *p*-value of 0.66 and an effect size of 0.06) and 4.4 to 2.8 for DLQI (with a *p*-value of 0.04 and an effect size of 0.31)). Patients also used the virtual assistant to do 52 medical consultations. Moreover, the usability is above average, with a SUS score of 70.1. As supported by MMAS-8 results, adherence also improved slightly. Our work demonstrates the improvement of the quality of life with the use of a virtual assistant in teledermatology, which could be attributed to the sense of security or peace of mind the patients get as they can contact their dermatologists directly within the virtual assistant-integrated system.

## 1. Introduction

Psoriasis is an immune-mediated disease that may cause visible signs of inflammation (e.g., raised plaques and scales on the skin) owing to inflammation caused in the body [1]. Psoriasis affects 125 million people worldwide (around 2 to 3 percent of the total population) [2]. Stress or anxiety, injury to the skin, hormonal changes, or certain infections or medications are factors that could trigger psoriasis flare-ups. Psoriasis can occur in any area of the body, such as hands, feet, nails, and the scalp [3].

The severity of psoriasis is measured based on the scales that assess physical symptoms, but it can also be measured by how the disease affects a person’s quality of life. Nearly 60% of patients with psoriasis communicated their disease as a large problem in their everyday lives [4]. Roughly one-quarter of people living with psoriasis have moderate to severe cases [5]. Furthermore, psoriatic patients with moderate to severe cases experienced a greater negative impact on their quality of life [6]. Psoriasis also generates stress derived from social stigma and altered body image. To sum up, psoriasis has a substantial psychological and social impact on patients.

Teledermatology generally uses apps and home-centered platforms for skin image monitoring and image sharing, where dermatologists can track patients’ responses to therapy or receive skin image information. The use of teledermatology helps review a large number of cases in a short time. Teledermatology also helps in the communication between specialists and patients. Better treatment outcomes are obtained when the communication between doctors and patients is improved [7]. The use of teledermatology seems to be gradually taking a central place in healthcare delivery [8]. Several apps exist for skin image monitoring, such as SymTrac™ Psoriasis [9], which stores photographs of the affected areas and tracks symptoms and quality of life over time; AI Psoriasis App: Manage and Care [10], which provides the severity rating of psoriasis from a skin image provided; and Imagine Skin Condition Tracker [11], an app that can track and compare photos over time to see the symptoms progress. There are also tools to improve psoriasis well-being like Kopa [12], which offers tips and tricks for handling symptoms; Claro [13], which helps enhance emotional well-being; and MiPsoriasis [14], which monitors psoriasis through questionnaires.

Virtual assistants (a.k.a. chatbots) are programs that perform tasks or services asked through text or voice-based conversations with users. Earlier bots in dermatology, such as Custom-RXBot [15], proposed bot prototypes to guide personalized medicament design. Sager et al. [16] analyzed the use of bots in health misinformation environments on Reddit’s dermatology forums. The bot posted prefabricated responses when misinformation was found. Former studies on improving medication adherence with virtual assistants or smartphone applications showed a positive effect on patient adherence [17,18,19]. Domogalla et al. [20] validated a disease management smartphone app for improving the mental health of patients with psoriasis in the long term. Furthermore, online care compared with in-person care showed equivalent improvements in disease severity among patients with psoriasis [21]. The improvement of quality of life for online and in-person care was also studied, which found that the online model and the in-person care had similar enhancement for psoriatic patients [22].

This study aims to build on the findings of these preliminary studies and investigate the impact on the quality of life of patients with psoriasis using a virtual assistant, which includes specific functionalities such as remote medical consultations with dermatologists and storage and viewing of patient photographs. The study also examines the usability and acceptance of the virtual assistant as well as the quality-of-life questionnaires and medication adherence scales to validate our virtual-assistant-integrated system.

## 2. Materials and Methods

### 2.1. Virtual Assistant

The scenario overview consists of a virtual assistant running remotely, connected with users through a messaging platform. The use of messaging platforms aligns with users’ daily use of their smartphones and provides a new communication channel to interact with healthcare services. The messaging platform used for this study was Signal [23], which is an end-to-end encrypted messaging platform that allows the use of chatbots. Therefore, the virtual assistant in our study is a messenger represented by a mobile phone number, which is added to users’ contact lists and is interacted with through Signal.

The core technological structure of the virtual assistant is described in our previous work [24,25]. A few adaptations by adding or modifying the functionalities were made due to the specific needs of the study. The data is stored in an encrypted server. Moreover, the database used to store medical data complies with the Health Level 7 Fast Healthcare Interoperability Resources (FHIR) clinical standard. However, the virtual assistant does not have integration with the hospital’s electronic medical record. The backups are securely encrypted too. Additionally, the patients are the only users able to view their confidential data. Any healthcare professional can only view the patients’ data if the patients explicitly let them see their data through Signal.

**Figure 1:**
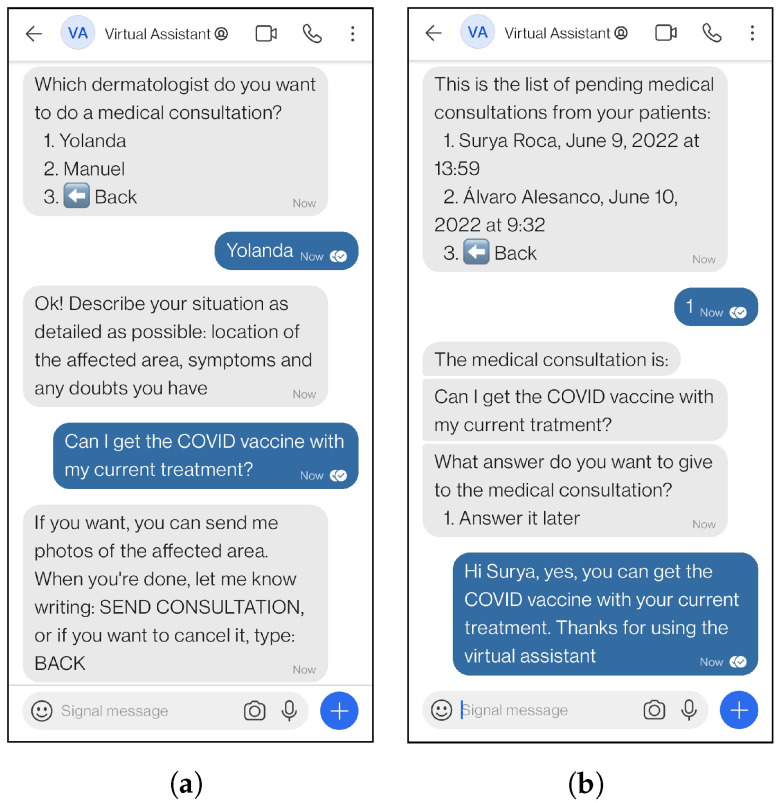
Example of real interactions with the virtual assistant using *Medical consultation* functionality. (**a**) Patient interaction. (**b**) Healthcare professional interaction.

**Figure 2:**
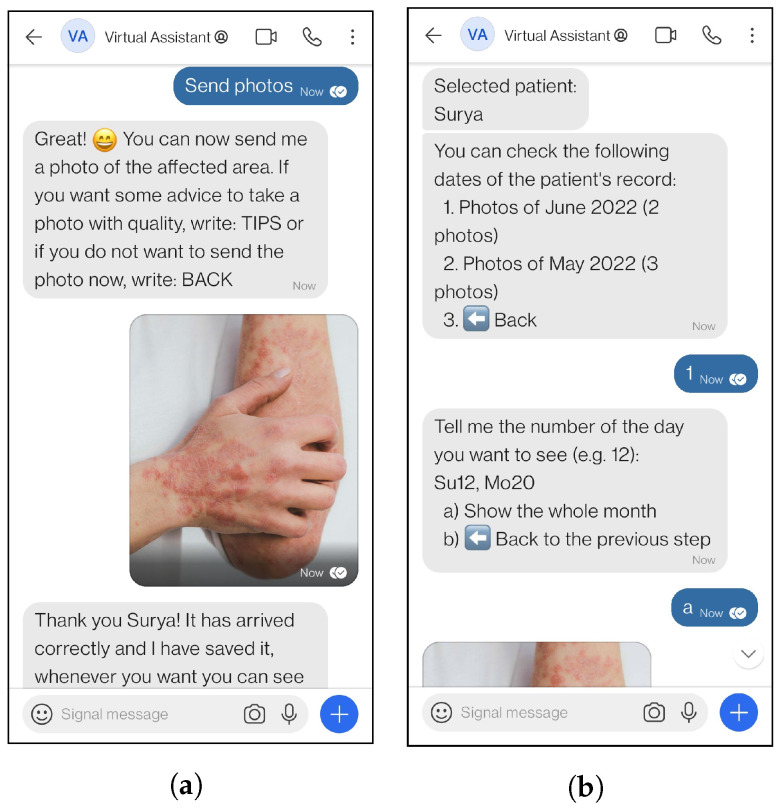
Example of real interactions with the virtual assistant. (**a**) Patient interaction using *Send photos* functionality. (**b**) Healthcare professional interaction using *Record* functionality.

Figure 1 shows the conversation between a patient and the virtual assistant while the patient is doing a medical consultation (Figure 1a) and the dermatologist answering the query through the virtual assistant (Figure 1b). Another example of how the system works is shown in Figure 2, where a patient is saving a photograph (Figure 2a), and the dermatologist is obtaining the saved photos of a patient through the virtual assistant (Figure 2b).

### 2.2. Virtual Assistant Functionalities

To be able to provide monitoring and connectivity services, the virtual assistant has several functionalities described in Table 1. The functionalities were discussed and modified according to the healthcare professionals’ needs throughout the study. The *Questionnaires* functionality provides services related to creating, modifying, exporting results, and setting reminders for healthcare professionals, and filling in and showing questionnaires for patients. The virtual assistant has a reminder option that periodically asks the patients to do the questionnaires. The healthcare professionals oversee those reminders and configure the frequency and the hour of the reminder. To provide communication between patients and healthcare professionals, the *Medical consultation* functionality was developed. Patients can, at any time, delete the medical consultations that they make. Moreover, the virtual assistant allows users to save and display patients’ photographs to monitor their affected area using *Send photos* and *Record* functionality (for example, the patient saves photographs every day during a flare-up in the *Send photos* functionality and later on sees the progress of the symptoms over time, thanks to the *Record* functionality).

### 2.3. Study Design

We designed and implemented a one-year prospective study with psoriatic patients and dermatologists to test the usage of a virtual assistant and its impact on the patient’s quality of life. The study was conducted from 22 April 2021 to 22 April 2022. The virtual assistant used in the system has two-fold usage: (1) as a tool to connect patients with healthcare professionals and vice versa (teledermatology); (2) as a monitoring tool for disease management (questionnaires, medication administration, and image records). We analyzed whether a virtual assistant could improve patients’ quality of life by using a virtual assistant that connects patients with healthcare professionals through online medical consultations.

Participants who met the inclusion criteria received detailed information about the study, their privacy, and anonymity, and were invited to participate in the study. A written and signed informed consent was obtained from all the participants (patients and healthcare professionals). The healthcare professionals did face-to-face personal interviews with the patients to include them in the study. In face-to-face medical consultations, the healthcare professionals explained to the patients how the virtual assistant works, helped them to download and install the messaging platform, and registered them. They also explained how a medical consultation can be performed through the virtual assistant.

### 2.4. Participants

Eligible participants were healthcare professionals and patients with psoriasis from the Miguel Servet University Hospital in Zaragoza, Spain. Healthcare professionals were 18 years old or older. The sample size included in the study was based on the availability of healthcare professionals and patients who met the requirements for inclusion criteria. Specifically, patients were recruited with the following inclusion criteria:

- Moderate–severe psoriasis under follow-up in the monographic psoriasis consultation (a consultation in which only one pathology is attended, in this case, patients with psoriasis), defined as one or more of the following points:
  — PASI (Psoriasis Area Severity Index) > 10;
  BSA (Body Surface Area) > 10;
  IGA (Investigator Global Assessment) scale levels 3 or 4;
  DLQI (Dermatology Life Quality Index) [26] > 5;
  Classic systemic treatment;
  Biological systemic treatment.
- Age between 18 and 65 years old;
- Patients can read and understand Spanish;
- Own a smartphone with Android or iOS and internet access;
- Patients have not used other telemedicine apps or chat-based care platforms before;
- The patient demonstrates a good level of technological knowledge and handling of smartphones (i.e., the patient needs to know how to use a smartphone and its apps with ease.). The technological knowledge was assessed based on the impression of healthcare professionals;
- Excluded from the study were subjects with cognitive, visual, or physical impairments that would interfere with the use of the virtual assistant and patients without a smartphone.

### 2.5. Outcomes Measures

One relevant parameter was the total number of medical consultations done. Moreover, another relevant parameter was the average number of photos per patient (who stored photographs in the system), which was calculated using the total number of photos sent and stored and the number of patients who used *Send photos* functionality to do so.

The answers to clinical questionnaires from all patients were evaluated before (at the beginning of the study) and after using the virtual assistant. The selected questionnaires were the standard and widely validated ones used in all pivotal trials of psoriasis drugs and other psoriasis studies. The percentage of questionnaires answered was also measured. Additionally, the number of patients active in answering the questionnaires was also obtained (a patient is considered active when the percentage of questionnaires answered is higher or equal to 50%). The selected questionnaires were:

- Psoriasis Quality of Life (PSOLIFE) [27]: PSOLIFE is a psoriasis quality of life questionnaire consisting of 20-item responses with a range from 20 to 100 points. Higher values of PSOLIFE score mean a better quality of life related to health (or less impact on the quality of life) [28];
- Dermatology Life Quality Index (DLQI): DLQI is a dermatological quality of life questionnaire consisting of 10-item responses with a range from 0 to 30 points. 0 means the patient does not have any problem and 30 means the illness in the patient has a severe impact;
- Treatment Satisfaction Questionnaire for Medication (TSQM) [29]: TSQM is a measure widely used to assess treatment satisfaction. TSQM scores on four different scales: effectiveness, side effects, convenience, and global satisfaction, each from 0 to 100. Higher values of TSQM scores mean higher satisfaction;
- Eight-item Morisky Medication Adherence Scale (MMAS-8) [30]: MMAS-8 is a widely used questionnaire that measures medication-taking behavior and consists of 8-item responses with a range from 0 to 8. It is measured using the following criteria: Items 1, 2, 3, 4, 6, and 7: Yes is 0 and No is 1. Item 5: Yes is 1 and No is 0. Item 8: Never/Rarely is 1, From time to time is 0.75, Sometimes is 0.5, Normally is 0.25 and Always is 0. A score of 8 reflects high adherence, values of 7 or 6 reflect medium adherence, and scores lower than 6 reflect low adherence.

In addition, some follow-up questionnaires were asked to observe the progress of their quality of life. These questionnaires were related to sleep, alcohol, and positiveness. Furthermore, to analyze the participants’ use of the virtual assistant, the number of messages sent to each functionality was measured.

Finally, a satisfaction survey was conducted with all participants in the study to obtain their opinion and satisfaction with the virtual assistant. We used the Spanish version [31] of the System Usability Scale (SUS) [32] complemented with some questions from the mHealth App Usability Questionnaire (MAUQ) [33] (a few items adapted from MAUQ Usefulness (MAUQ_U)). The SUS score range is from 0 to 100. The results are considered above average when the SUS score is above 68, and results below 68 are below average. The final version of the surveys can be accessed in Multimedia Appendix A and Appendix B. At the end of the study, the participants received the link to the satisfaction survey, and after a few days, the healthcare professionals made phone calls to remind them about the importance of answering the satisfaction survey. The surveys were anonymized once they were obtained to increase honesty and decrease bias. The answers with multiple options for the participants’ satisfaction survey were weighted with the following scale: totally agree (7), quite agree (6), somewhat agree (5), neutral (4), somewhat disagree (3), quite disagree (2), and totally disagree (1); too easy (5), easy (4), somewhat easy (3), difficult (2) and too difficult (1); always (5), almost always (4), sometimes (3), rarely (2), and never (1).

### 2.6. Ethical Aspects

The study provides the required measures of privacy and users’ rights by complying with both national data protection law LO 03/2018 [34] and European General Data Protection Regulation (GDPR) [35]. The study protocol was approved and registered by the Comité de Ética de la Investigación de la Comunidad Autónoma de Aragón (CEICA) [36] on 7 October 2020 (minutes nº 19/2020). The CEICA committee acts in accordance with the Declaration of Helsinki (last modified in 2013) and with the Good Clinical Practice (GCP) standard.

### 2.7. Statistical Analysis

We used frequency and percentage to describe categorical variables while for the continuous variables, we used mean and standard deviation (SD). The effect size was calculated using Cohen’s D. To check the normality of the data, the Shapiro–Wilk normality test was used. The normality can be assumed when the *p*-value is more than 0.05. To compare the outcomes at the beginning and after using the virtual assistant, the Paired Samples T-test and Wilcoxon signed-rank test were estimated where appropriate for continuous variables (PSOLIFE, DLQI, TSQM, and MMAS-8 questionnaires, and satisfaction survey). Variables measured at the beginning and after were considered significantly different when the *p*-value was less than 0.05. All statistical analyses were conducted using R software, version 4.0.3 [37].

## 3. Results

### 3.1. Participants

**Figure 3:**
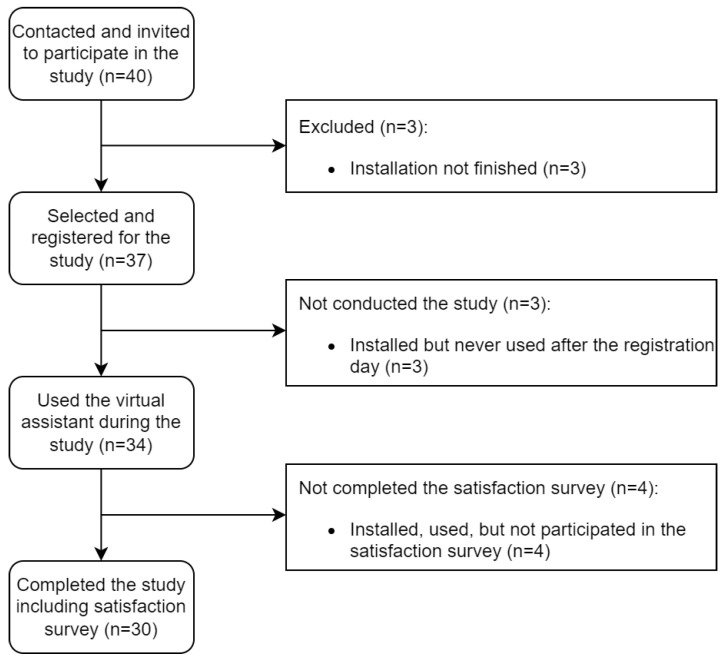
Patient selection, exclusion, and completion of the study.

During the study, four professionals from the department of dermatology used the virtual assistant (three dermatologists and one nurse). Throughout the year, the healthcare professionals contacted 40 patients and invited them to use the virtual assistant (as shown in Figure 3). Three patients did not finish the process of installing the messaging platform and registering themselves with the virtual assistant. After the selection and registration, three of them did not continue using the virtual assistant after the first day. A total of 34 psoriatic patients used the virtual assistant (with at least 4 months of usage for the last patient registered in the virtual assistant). Finally, 30 patients completed the final satisfaction survey. The composition of patients was 36.7% females (11/30) and 63.3% males (19/30). The average age of patients was 36.0 years old (SD 10.0) with a range from 18 to 58 years old. The healthcare professionals were 50% females (2/4) and 50% males (2/4). The average age of healthcare professionals was 35.3 years old (SD 7.0) with a range from 27 to 44 years old.

### 3.2. Evaluation Outcomes

Patients used the virtual assistant for a total of 52 remote medical consultations with healthcare professionals. Moreover, patients stored 29 photos in the virtual assistant. The photos were stored by 13.3% of the patients (4/30) involved in the study, with an average of 7.3 photos (SD 5.4) by the patient who saved photographs.

The results of the questionnaire are shown in Table 2 (results are not significantly different). A total of 51.1% (284/556) of the questionnaires were answered by the patients. A total of 60.0% of patients (18/30) were active in answering the questionnaires. Average scores improved for PSOLIFE (from 63.8 to 64.8), DLQI (from 4.4 to 2.8), TSQM: Convenience (from 74.8 to 79.1), TSQM: Global satisfaction (from 63.7 to 68.5) and MMAS-8 (from 6.9 to 7.2). The first MMAS-8 questionnaire was asked on 20 October 2021, 6 months after the start of the study. The last MMAS-8 questionnaire was asked on 18 April 2022. Nevertheless, TSQM: Effectiveness (from 61.8 to 61.4) and TSQM: Side effects (from 25.7 to 18.4) show a decrease in the scores after patients used the virtual assistant.

The follow-up questionnaire results are shown in Table 3. The percentage of follow-up questionnaires answered was 59.9% (358/598). For the follow-up questionnaires, a total of 66.7% of patients (20/30) were actively answering them. We did not collect a baseline measurement for sleep, alcohol, or positiveness. The question to follow up on the sleep was “Did you sleep well today?” and 70.1% of the patients answered “Yes”. Furthermore, the answer was “Nothing” in 74.6% of answers regarding alcohol intake. The average for the positiveness questionnaire was 3.8 (SD 1.0) (with the question “How do you think your day will go today? Range from 0 (very bad) to 5 (very good)”).

The analysis of the functionalities used by participants is shown in Table 4. The table shows the percentage of messages sent by participants to the virtual assistant to observe the real usage of functionalities and avoid the notification messages related to questionnaires that are not answered (and therefore not used). We normalized the data by dividing the messages sent to each functionality by the total of messages sent by a participant. After that, we did the average of the normalized messages of all the participants. The results show that *Questionnaires* functionality is the main functionality used (78.0%), followed by *Medical consultation* functionality with 19.8%.

### 3.3. Participant Satisfaction Survey

The first outcome observed in the satisfaction survey is the SUS score of the participants of the study. The average SUS score is 70.1 (SD 15.2) (range of SUS score from 0 to 100). The outcomes from the satisfaction survey divided by patients and healthcare professionals are detailed in the following subsections.

#### 3.3.1. Patients’ Survey Outcomes

The outcomes related to the patients’ answers are shown in Table 5 and Table 6. More than half of the patients (18/30) agree that *Medical consultation* functionality is the option they like most. *Send photos* functionality was chosen by 10% (3/30) of the patients. The rest of the patients chose other functionalities related to medication and appointments. One patient liked all the functionalities. Furthermore, patients agreed that it was easy (with an average of 4.3 (SD 1.0)) to answer questionnaires through the virtual assistant. Moreover, the face-to-face medical appointments attendance before and after using the virtual assistant decreased from 4.7 (SD 0.8) to 4.5 (SD 1.1), with an effect size of 0.21 (the result is not significantly different with *p* = 0.27).

The main reasons why patients left questionnaires unanswered were: I read the notification but then I forgot to answer (11/30), I don’t have time (7/30), too long/too many/too monotonous (6/30), and I was not aware of the mobile notification (4/30).

Finally, some comments noted from patients were: “I use it frequently if I have a problem with my biological therapy, I find it extraordinary to be attended to at any time.”, “Right now my psoriasis is under control but it will come in handy when I have a flare-up.”, “If my psoriasis gets worse and I need a close follow-up, I would use the virtual assistant more.”, and “I believe that it is a very necessary tool. The psoriasis condition can vary a lot from one medical consultation to another and it gives a lot of security to have it and to be able to consult the doctor or nurse if there is any mishap, doubt, or flare-up at that time without having to wait until the appointment arrives. It helps to have the disease more controlled. I would recommend it 100%.”.

#### 3.3.2. Healthcare Professionals’ Survey Outcomes

The outcomes related to the healthcare professionals’ answers are shown in Table 7 and Table 8. The functionality the healthcare professionals agree that they like the most is *Medical consultation*. Furthermore, the average score regarding how easy or difficult they find the usage of the *Questionnaires* functionality (generating or assigning questionnaires) was 3.8 (SD 0.5).

## 4. Discussion

### 4.1. Principal Results

New messaging-based mobile phone systems for chronic diseases have been deployed, but such systems lack evaluation. Further research on evaluating mHealth interventions and their user acceptance should be addressed [38]. This study attempted to address the gap by conducting a one-year prospective study to evaluate the use of a virtual assistant in teledermatology. The virtual assistant is a complex and comprehensive system that facilitates daily psoriasis monitoring and patient-healthcare professional medical consultations.

The key findings highlighted the improvement in the patient’s quality of life and the good usability of the virtual assistant. The improvement in the quality of life is demonstrated by higher PSOLIFE scores (with a mean difference of 1) and lower DLQI scores (mean difference of 1.6). These results agree with the findings of a study conducted by Kornmehl et al. [39], which found that DLQI scores increased by 4.1 using online management for atopic dermatitis after 12 months. The adherence also improved slightly (with a mean difference of 0.3), which may be due to the fact that patients pay attention to a tool related to their disease, and, therefore, they may pay more attention to the treatment. The availability of a physician anytime they require could have also helped. This result is consistent with the study by Rhee et al. [40], which observed improvement in the awareness of symptoms and treatment adherence and sense of control with the use of their system. The result is also consistent with the answer in the survey related to treatment adherence, where 66.7% of patients (20/30) think the virtual assistant improved their adherence. Additionally, the above average (with a value of 70.1, above 68) SUS score obtained for the usability of the virtual assistant is consistent with the scores obtained for similar systems described in Refs. [41,42,43].

Only half of the patients (15/30) did consultations with their healthcare professionals through the virtual assistant. This result may be due to the fact that psoriasis is a disease based on flare-ups that are not regular and can or cannot appear in the patients during the one-year study period. Furthermore, patients stored 29 photos in the virtual assistant to save their progress (they sent photos of their psoriasis plaques, their nails, their hair, etc.). During the consultation, physicians could see the date on which the photographs were taken and assess the disease evolution.

### 4.2. Patient Acceptance

The patients seemed to agree to the use of the virtual assistant as a total of 73.3% of patients (22/30) agreed to the idea of using a virtual assistant even after the project (Table 6). This outcome is consistent with the findings observed by Nadarzynski et al. [44], where the acceptability of chatbots in healthcare was 67%. Another key point to emphasize is that 83.3% of the patients (25/30) found having a tool to contact their dermatologist provides them with a sense of security or peace of mind as the virtual assistant enables them to consult the doctor any time between in-person consultations.

A total of 66.7% of patients (20/30) were active in answering the follow-up questionnaires, in contrast to 60.0% of patients (18/30) active in answering the clinical questionnaires (PSOLIFE, DLQI, etc.). The follow-up questionnaires were shorter than clinical questionnaires, which may explain why patients were more involved in answering them (indeed, 6 out of 30 patients agreed with the idea that the questionnaires were too long, too many, or too monotonous).

We observed that face-to-face medical consultations were not reduced in general (only 16.7% of patients (5/30) reduced the number of consultations after using the virtual assistant). This result is consistent with the idea discussed by Corbett et al. [45], which views telemedicine as an adjunct to in-person consultations, unable to replace them.

### 4.3. Healthcare Professional Acceptance

All the healthcare professionals agree with the idea of using the virtual assistant after the project. Moreover, a total of 75% of healthcare professionals agree with the idea that it is easy to answer patient queries. Answering patient queries is crucial because if a physician cannot answer their questions not knowing how to use it, the patient could feel frustrated, start feeling mistrust, and stop using the virtual assistant.

Another essential factor is the quality of the images. When an image is sent through a messaging platform, it is compressed, reducing the original quality of the image. The images should have enough quality to allow dermatologists to observe the disease conditions and make decisions accordingly. In the survey, all the healthcare professionals answered that the quality of the images was sufficient to make diagnoses. Thus, virtual assistant-integrated systems could also be used for other diseases such as dermatological affections, which need image support.

### 4.4. Limitations

This prospective study has several limitations. The reduced number of dermatologists participating in the study does not allow us to obtain statistically significant differences in the results. Therefore, further studies with more dermatologists are needed to generalize the results. In addition, the sample size was small, which should be extended to cover more samples in the future.

Sometimes patients need a dermatological examination that includes palpation, dermatoscopy, microbiology studies, Wood lamp examination, or biopsy (for example, when the disease or new conditions are diagnosed). Such an examination is not currently possible with the virtual assistant. A face-to-face consultation is required in such cases. Additionally, patients not providing enough photographs to dermatologist during remote medical consultation has risks of misdiagnosis (e.g., other sites affected, atypical presentation, or clinical lookalikes).

The integration of new methods for healthcare professionals has burnout risks [46]. So, training of such methods should be conducted within their working hours. Our system allows healthcare professionals to set the virtual assistant notifications in silent mode while they are not working so that medical consultations are managed only while at work.

Furthermore, the patients’ usability regarding medical consultations is closely related to the psoriasis flare-ups. As observed in the comments made by patients, some did not have outbursts during the study period, but, notwithstanding, they consider it will be helpful when they have a flare-up in the future. Moreover, the participants need to have digital literacy to use messaging platforms or have people who can help them if they find difficulties.

## 5. Conclusions

This study evaluated the use of virtual assistants in teledermatology, specifically, to connect patients with dermatologists remotely using a messaging platform. During the evaluation, the study provided an overall perspective of how the patient’s quality of life is affected by the use of virtual assistants. The general usability of the virtual assistant was above average (with a SUS score of 70.1), and 26 out of 34 participants agreed to continue using the virtual assistant after the study. The patient’s quality of life improved (with a mean difference of 1 and 1.6 for PSOLIFE and DLQI, respectively). Furthermore, our results suggest that virtual assistants can provide a tool to improve patient’s treatment adherence (agreed by 66.7% of the patients). The use of virtual assistant also provided security or peace of mind (83.3% of the patients agreed with it) to patients as they could directly contact dermatologists.

In the future, we will consider the integration of the virtual assistant with the hospital’s electronic medical record and using it for other chronic diseases. We will also conduct detailed evaluations of long-term effects on healthcare professionals, such as the possible burnout created by the incorporation of virtual assistants to their working time.

## Figures and Tables

**Table 1:** Description of the virtual assistant functionalities used in the study.

Functionality	Tasks
Questionnaires	1. Create
	2. Modify
	3. Show
	4. Delete
	5. Fill in questionnaires
	6. Set reminders related to filling in the questionnaire
	7. Export results
Medical consultation	1. Redirect the questions that patients have about their illnesses to the
	dermatologists
	2. Save the answers of the dermatologists to the queries
	3. Show the answers to the patients
	4. Modify the answers to medical consultations
	5. Delete medical consultations
Send photos	1. Save patient’s photographs
	2. Explain how to take a good photograph
Record	Display the images that are saved in the virtual assistant

**Table 2:** Average and SD of the first and last PSOLIFE, DLQI, TSQM, and MMAS-8 answered questionnaires.

Questionnaire [Range]	First Time Filled, Average (SD ^5^)	Last Time Filled, Average (SD ^5^)	*p*-Value	Effect Size
PSOLIFE ^1^ [20–100]	63.8 (16.9)	64.8 (15.4)	0.66	0.06
DLQI ^2^ [0–30]	4.4 (4.9)	2.8 (5.1)	0.04	0.31
TSQM ^3^: Effectiveness [0–100]	61.8 (29.2)	61.4 (26.2)	0.95	0.01
TSQM ^3^: Side effects [0–100]	25.7 (33.4)	18.4 (30.1)	0.31	0.23
TSQM ^3^: Convenience [0–100]	74.8 (24.7)	79.1 (20.4)	0.43	0.19
TSQM ^3^: Global satisfaction [0–100]	63.7 (24.4)	68.5 (18.6)	0.66	0.22
MMAS-8 ^4^ [0–8]	6.9 (1.5)	7.2 (0.7)	0.57	0.28

^1^ Psoriasis Quality of Life; ^2^ Dermatology Life Quality Index; ^3^ Treatment Satisfaction Questionnaire for Medication; ^4^ Eight-item Morisky Medication Adherence Scale; ^5^ Standard deviation.

**Table 3:** Results related to the follow-up questionnaires (sleep, alcohol, and positiveness).

Follow-Up Questionnaire	Question	Answers
Sleep	“Did you sleep well today?”	70.1% (117/167) of answers were “Yes”
Alcohol	“How much alcohol did you drink today?”	74.6% (47/63) of answers were “Nothing”
Positiveness	“How do you think your day will go today? Range from 0 (very bad) to 5 (very good)”	Average of 3.8 (SD ^1^ 1.0)

^1^ Standard deviation.

**Table 4:** Participants’ usage analysis of the functionalities.

Functionality	Normalized Number of Messages Sent by Participants, % (*n*) (*N* = 34)
Questionnaires	78.0 (26.5)
Medical consultation	19.8 (6.7)
Record	1.4 (0.5)
Send photos	0.7 (0.2)

**Table 5:** Patients’ responses regarding the virtual assistant utility.

Affirmations [Range from 1 to 7]	Mean (SD ^1^)
The language used by the virtual assistant was appropriate.	6.0 (1.3)
The virtual assistant was useful to improve my quality of life.	4.8 (1.7)
The virtual assistant made it convenient for me to communicate with my health	5.5 (1.7)
care provider.	
I felt confident that any information I sent to my provider using the virtual	5.4 (1.5)
assistant would be received.	
I felt comfortable communicating with my health care provider using the virtual	5.4 (1.6)
assistant.	

^1^ Standard deviation.

**Table 6:** Patients’ responses regarding general use and perception of the virtual assistant.

Question	Yes, % (*n*/*N*)	No, % (*n*/*N*)
Did you do medical consultations with your dermatologist	50.0 (15/30)	50.0 (15/30)
through the virtual assistant?		
If you did medical consultations, were they resolved	80.0 (12/15)	20.0 (3/15)
satisfactorily?		
Has having a tool with which to be able to contact your		
dermatologist directly provided you with security/peace of	83.3 (25/30)	16.7 (5/30)
mind?		
Do you think the virtual assistant improves your treatment	66.7 (20/30)	33.3 (10/30)
adherence?		
Do you stop using the virtual assistant when you feel better with	23.3 (7/30)	76.7 (23/30)
your symptoms?		
Have you reduced the number of face-to-face medical	16.7 (5/30)	83.3 (25/30)
consultations after using the virtual assistant?		
Do you think you will continue using the virtual assistant after	73.3 (22/30)	26.7 (8/30)
the project?		

**Table 7:** Healthcare professionals’ responses regarding the virtual assistant utility.

Affirmations [Range from 1 to 7]	Mean (SD ^1^)
The language used by the virtual assistant was appropriate.	5.8 (0.5)
The virtual assistant was useful to improve the quality of life of my patients.	6.0 (0.8)
The virtual assistant made it convenient for me to communicate with my patients.	6.3 (1.0)
I felt confident that any information I sent to my patients using the virtual	5.5 (0.6)
assistant would be received.	
I felt comfortable communicating with my patients using the virtual assistant.	5.5 (1.0)

^1^ Standard deviation.

**Table 8:** Healthcare professionals’ responses regarding general use and perception of the virtual assistant.

Question	Yes, % (*n*) (*N* = 4)	No, % (*n*) (*N* = 4)
Do you think the virtual assistant improves the patients’ treatment adherence?	75.0 (3)	25.0 (1)
Was the quality of the images sufficient to make diagnoses?	100.0 (4)	0.0 (0)
Was it easy for you to answer patients’ queries?	75.0 (3)	25.0 (1)
Have you seen the number of face-to-face medical consultations of patients reduced after using the virtual assistant?	100.0 (4)	0.0 (0)
Do you think you will continue using the virtual assistant after the project?	100.0 (4)	0.0 (0)

## Abbreviations

The following abbreviations are used in this manuscript:

BSA	Body Surface Area
CEICA	Comité de Ética de la Investigación de la Comunidad Autónoma de Aragón
DLQI	Dermatology Life Quality Index
FHIR	Fast Healthcare Interoperability Resources
GDPR	General Data Protection Regulation
IGA	Investigator Global Assessment
MMAS-8	Eight-item Morisky Medication Adherence Scale
PASI	Psoriasis Area Severity Index
PSOLIFE	Psoriasis Quality of Life
SD	Standard deviation
TSQM	Treatment Satisfaction Questionnaire for Medication

## Appendix A. Patient’s Satisfaction Survey

Name:

Surname:

For each statement, please choose the best option that suits your experience with the virtual assistant.

Virtual assistant usability:

**AFFIRMATIONS**	**Strongly Agree**	**Agree**	**Neutral**	**Disagree**	**Strongly Disagree**
I think that I would like to use this virtual assistant frequently					
I found the virtual assistant unnecessarily complex					
I thought the virtual assistant was easy to use					
I think that I would need the support of a technical person to be able to use this virtual assistant					
I found the various functions in this virtual assistant were well integrated					
I thought there was too much inconsistency in this virtual assistant					
I would imagine that most people would learn to use this virtual assistant very quickly					
I found the virtual assistant very cumbersome to use					
I felt very confident using the virtual assistant					
I needed to learn a lot of things before I could get going with this virtual assistant					

Virtual assistant utility:

**AFFIRMATIONS**	**Totally Agree**	**Quite Agree**	**Somewhat Agree**	**Neutral**	**Somewhat Disagree**	**Quite Disagree**	**Totally Disagree**
The language used by the virtual assistant was appropriate							
The virtual assistant was useful to improve my quality of life							
The virtual assistant made it convenient for me to communicate with my health care provider							
I felt confident that any information I sent to my provider using the virtual assistant would be received							
I felt comfortable communicating with my health care provider using the virtual assistant							

What option/feature do you like most about the virtual assistant?

❍ Consultations
❍ Questionnaires
❍ Medication
❍ Send photos
❍ Medical appointments
❍ Other:

Questionnaires:

**QUESTION**	**Too Easy**	**Easy**	**Somewhat Easy**	**Difficult**	**Too Difficult**
How easy or difficult was it for you to answer the questionnaires in the virtual assistant?					

What were the main reasons why you left questionnaires unanswered?

❍ I don’t have time
❍ Too monotonous
❍ Too long
❍ I did not consider that they were necessary for the psoriasis follow-up
❍ I read the notification but then I forgot to answer
❍ I was not aware of the mobile notification
❍ Other:

Face-to-face medical appointments:

**QUESTION**	**Always**	**Almost Always**	**Sometimes**	**Rarely**	**Never**
Did you attend face-to-face medical appointments BEFORE using the virtual assistant?					
Do you attend face-to-face medical appointments AFTER using the virtual assistant?					

Did you do medical consultations with your dermatologist through the virtual assistant?

❍ Yes
❍ No

If you did medical consultations, were they resolved satisfactorily?

❍ Yes
❍ No

Has having a tool with which to be able to contact your dermatologist directly provided you with security/peace of mind?

❍ Yes
❍ No

Do you think the virtual assistant improves your treatment adherence?

❍ Yes
❍ No

Do you stop using the virtual assistant when you feel better with your symptoms?

❍ Yes
❍ No

Have you reduced the number of face-to-face medical consultations after using the virtual assistant?

❍ Yes
❍ No

Do you think you will continue using the virtual assistant after the project?

❍ Yes
❍ No

Do you have any suggestions to improve the virtual assistant?

What would encourage you to use the virtual assistant more often?

Any other comment

Thank you very much for participating! Your opinion is very important for us!

## Appendix B. Healthcare Professional’S Satisfaction Survey

Name:

Surname:

For each statement please choose the best option that suits your experience with the virtual assistant.

Virtual assistant usability:

**AFFIRMATIONS**	**Strongly Agree**	**Agree**	**Neutral**	**Disagree**	**Strongly Disagree**
I think that I would like to use this virtual assistant frequently					
I found the virtual assistant unnecessarily complex					
I thought the virtual assistant was easy to use					
I think that I would need the support of a technical person to be able to use this virtual assistant					
I found the various functions in this virtual assistant were well integrated					
I thought there was too much inconsistency in this virtual assistant					
I would imagine that most people would learn to use this virtual assistant very quickly					
I found the virtual assistant very cumbersome to use					
I felt very confident using the virtual assistant					
I needed to learn a lot of things before I could get going with this virtual assistant					

Virtual assistant utility:

**AFFIRMATIONS**	**Totally Agree**	**Quite Agree**	**Somewhat Agree**	**Neutral**	**Somewhat Disagree**	**Quite Disagree**	**Totally Disagree**
The language used by the virtual assistant was appropriate							
The virtual assistant was useful to improve the quality of life of my patients							
The virtual assistant made it convenient for me to communicate with my patients							
I felt confident that any information I sent to my patients using the virtual assistant would be received							
I felt comfortable communicating with my patients using the virtual assistant							

Do you think the virtual assistant improves the patients’ treatment adherence?

❍ Yes
❍ No

Was the quality of the images sufficient to make diagnoses?

❍ Yes
❍ No

What option/feature do you like most about the virtual assistant?

❍ Answer consultations
❍ Questionnaires
❍ Medication
❍ Send photos
❍ Medical appointments
❍ Send message
❍ Psoriasis scale
❍ Other:

Questionnaires:

**QUESTION**	**Too Easy**	**Easy**	**Somewhat Easy**	**Difficult**	**Too Difficult**
If you generated or assigned questionnaires, how easy or difficult was it for you?					

Was it easy for you to answer patients’ queries?

❍ Yes
❍ No

Have you seen the number of face-to-face medical consultations of patients reduced after using the virtual assistant?

❍ Yes
❍ No

Do you think you will continue using the virtual assistant after the project?

❍ Yes
❍ No

Do you have any suggestions to improve the virtual assistant?

What would encourage you to use the virtual assistant more often?

Any other comment

Thank you very much for participating! Your opinion is very important for us!
